# The Use of Pill Counts as a Facilitator of Adherence with Antiretroviral Therapy in Resource Limited Settings

**DOI:** 10.1371/journal.pone.0067259

**Published:** 2013-12-05

**Authors:** Loice Achieng, Helen Musangi, Katherine Billingsley, Sharon Onguit, Edwin Ombegoh, LeeAnn Bryant, Jonathan Mwiindi, Nathaniel Smith, Philip Keiser

**Affiliations:** 1 A.I.C. Kijabe Hospital, Kijabe, Kenya; 2 University of Nairobi, Nairobi, Kenya; 3 University of Texas Medical Branch, Galveston, Texas, United States of America; 4 Arkansas Department of Health, Little Rock, Arkansas, United States of America; University of Toronto, Canada

## Abstract

**Background:**

Pill counts are often used to measure adherence to ART, but there is little data on how they affect adherence. We previously showed a bivariate relationship between clinicians counting pills and adherence in patients receiving HIV care in Kenya. We present a secondary analysis of the relationship between numbers of pill counts and clinical outcomes in resource limited settings

**Methods:**

Patients initiating ART at Kijabe Hospital were monitored for the number of discretionary pill counts performed by their clinician in the first 6 months of ART. Subjects were followed for at least 1 year after enrollment. The number of clinician pill counts was correlated to ART adherence. The primary endpoints were time to treatment failure, defined as a detectable HIV-1 viral load, death; or loss to follow-up.

**Results:**

Clinician pill counts were done at 68% of clinic visits for 304 subjects. There was a positive correlation between the number of clinician pill counts and ART adherence (*r* = 0.21, *p* <0.001). Patients were divided into 3 groups (0 counts, 1 to 3 counts, 4 to 7 counts) and exhibited adherence of 76%, 84%, and 92%, respectively (*p* = 0.004). Time to treatment failure for these groups was 220 days, 438 days, and 497 days (P<0.01), respectively. Time to virologic failure in living patients remaining in the cohort was longer in those with more pill count (P =0.02). Multi-variate analysis adjusting for co-variates affecting time to treatment failure found that that clinician pill counts were associated with a decreased risk of treatment failure (HR = 0.69, *p* =0.04).

**Conclusions:**

The number of clinician pill count performed was independently associated with better adherence and a decreased risk of treatment failure. The use of clinician pill counts should be further studied as an adherence promoter through a randomized clinical trial.

## Introduction

Adherence to antiretroviral therapy (ART) is an essential part of successful treatment programs. Without high adherence rates, it is impossible to realize the true benefits of therapy; benefits such as improved morbidity and mortality, decreased development of resistant virus, decreased transmission of virus, and improved quality of life for individuals infected with HIV[1,2]. A number of evidence based interventions to retain patients and foster adherence to medications have been used in sub-Saharan Africa, including: intensive patient counseling[3], community health worker[4], directly observed therapy [5], peer health workers [6], alarms and devices[7,8] , treatment buddies[9] and SMS texting[10]. Adherence can be measured directly through directly observed therapy; however it is often unfeasible or undesirable because of stigma. Because of this, adherence markers such as pill counts are often used as an outcome measure in these studies [7,8]. 

However, the potential exists for pill counting to mediate adherence outcomes. This may have both important clinical and research implications. Sustaining HIV therapy requires the identification and validation of cost effective methods to improve treatment outcomes. It is possible that counting pills is an easy way to foster better clinical results in patients taking HIV medication. However, any putative benefit to counting pills may also effect the interpretation of other intervention designed to improve patient’s adherence to HIV medication. Many clinical trials use pill counts as a metric assess adherence. Thus it would be important for future studies to understand how counting pills may affect outcome being studied. It is unknown what affect pill counting may have on patient adherence in resource limited settings. 

We had previously performed a prospective observational study to evaluate the determinants of retention into care and long term virologic suppression at a large HIV treatment program in central Kenya [11]. In this analysis we found a strong association between ever having a pill count performed by the clinical provider and treatment. However, our analysis in that study was limited to only determining whether a pill count was or was not done (yes or no). We found that pill counts were performed in only 68% of clinic visits. To further determine if pill counts are associated with better clinical outcomes, it would be important to know if adherence and clinical outcomes improved as patients had more clinician pill counts performed during their clinic visits. In the current study, we further analyze data from the observational cohort to determine if there is a quantitative relationship between clinician pill counts and clinical outcomes.

## Methods

The setting, study design and participants in the study have been previously described [11]. An overview is provided below. 

### Setting

AIC Kijabe Hospital is a 265 bed hospital in Central Kenya that follows approximately 6000 patients on ART within a 40 km radius of its clinics. Care is provided at the main hospital and at five satellite clinics. It uses a community centered, team based approach that includes intensive patient education, home visits, and support groups. Medications are dispensed by a Kijabe pharmacist. Medical records are abstracted and entered into an electronic database. Care is provided at the main hospital and at five satellite clinics. Monthly clinic visits are required and include a medical evaluation, unannounced pill counts, and pharmacy counseling.

#### Pill Counts

The Kijabe adherence program includes home visits and pharmacy counseling, both of which including a mandatory pill count by either a community health worker or a pharmacist. The program recommends that clinicians perform pill counts at each visit. During the clinic visits, clinicians are trained to count the number of anti-retroviral medications left in the pill bottle and to counsel the patient about adherence if the patient missed dosages. The provider writes the result of the pill count on the clinic encounter form next to the vital signs. There is no prompt or designated place for recording the pill count on the form. Patients would not know if their provider would count the pills on any particular visit. There are no incentives for performing pill counts or penalties for failing to do so. Similarly, there is no incentive or penalty for providers whose patients may or may not have better outcomes. As consequence the practice is left to the discretion of the provider. This study focuses only on the role of clinician pill counts and excluded pill counts performed by pharmacists and community health workers as these were not associated with outcomes in the previous study [11].

### Participants

Study participants were recruited from the Kijabe HIV program to join a prospective observational cohort to evaluate existing facilitators of adherence and treatment success. There was no intervention. To be eligible for inclusion, participants met the following criteria: 1) HIV positive, 2) Age greater than 18 years old, 3) Anti-retroviral naïve with the exception of women who had previously received medication as part of prevention of mother to child transmission, 4) Completion of anti-retroviral treatment preparation course, 5) Signed treatment contract, and 6) Providing informed consent.

### Data collection

Demographic data was collected at baseline, all other data was abstracted from patient charts. Baseline staging was completed using CD_4_ counts, (FACSCount, BD UK) per standard of care. Virologic failure was measured by HIV RNA viral loads at baseline, 6 months, and after 12 months of therapy using ExaVir HIV-1 RT assay (Cavidi, Uppsala Sweden). 

### Statistical analysis

The primary endpoint of the study was the time to treatment failure. Treatment successes were defined as alive, on anti-retroviral medication, with an HIV-1 below the level of detection. Anyone not meeting this definition was considered a treatment failure. All subjects were followed for at least one year. Subjects were considered lost to follow up if there was no contact after 90 days. All other categories were considered treatment failures including those subjects with a detectable viral load, those who died and those who were lost to follow up. The primary analysis was done on all patients who were enrolled into the study. A secondary analysis was performed on those patients who were alive and in care at the end of the study because bias for worse outcomes in those who died or who were lost to follow up.

In the primary analysis, participants were stratified by the number of physician pill counts received in six months: none, 1 to 3 pill counts, or more than 3 pill counts. Differences in baseline characteristics by pill count category were determined using ANOVA or CHI squared test where appropriate. Adherence to medication was collected by review of pharmacy refill records for each participant using the method described by El-Khatib [12]. In this method percentage adherence is calculated by dividing the number of daily dosages dispensed by the total number of days between start and stop dates on drugs. Linear regression was completed for adherence outcomes by number (total) of pill counts to assess for association. Tests of multi-linearity between covariates was performed the variance inflation factor (vif). Time to treatment failure was assessed by Kaplan-Meier survival analysis. Cox regression multivariate analyses were used to analyze overlapping effects of variables on outcomes and pill count category. Potential covariates for inclusion into the Cox model were first tested by univariate analysis. Those that were statistically significant were included into the final model. 

### Ethics

This study was approved by review of the AIC Kijabe Hospital Ethics Review Committee and the Internal Review Board at the University of Texas Medical Branch.

## Results

### Cohort description

There were 351 subjects screened and of those 301 subjects were enrolled. The fifty patients who screened out were children who were not eligible by according to IRB approval Ten subjects were transferred out of care and their data was censored. Of the remaining 291 subjects, 85 were treatment failures and 206 were treatment successes. Of the treatment failures, 48 (15.9 %) virologic failures, 15 deaths (5%) and 22 (7.9) were lost to follow up. There were 1236 un-announced pill counts performed by clinical providers during 1818 clinic visits for a pill count rate of 68%. 

Table 1 shows a baseline comparison between the groups defined as having no pill counts, 1 to 3 pills counts, or more than 3 pills counts in six months. There were no differences between the groups with regard to age, baseline viral load, employment status or distance to clinic at initiation of therapy, or clinic site where care was performed. CD4 counts were lowest in those patients who had pill counts performed greater than 3 times in a six month period. 

**Table 1:** Significant change in program variables (means) by number of pill counts.

**Factor**	**None (n=73)**	**1 to 3 counts (n=114)**	**> 3 counts (n=104)**	**P value**
Gender (% female)	8.8	33.4	58.2	<0.01
Baseline CD4 (mean)	234.7	257.83	176.01	0.01
Baseline viral load _log_ (copies)	5.19	5.04	5.01	0.32
Distance to clinic (km)	10.87	12.88	14.97	0.20
Support groups attended (median)	0.3	2.1	3.0	<0.01
Pharmacy Counseling Sessions (median)	0.2	1.1	1.1	<0.01
Home visits (median)	0.1	1.1	1.3	<0.01
Clinic visits (median)	1	5	6.1	<0.01
Viral load undetectable (% achieved)	66.7	77.1	84.1	0.20

### Adherence

**Figure 1:**
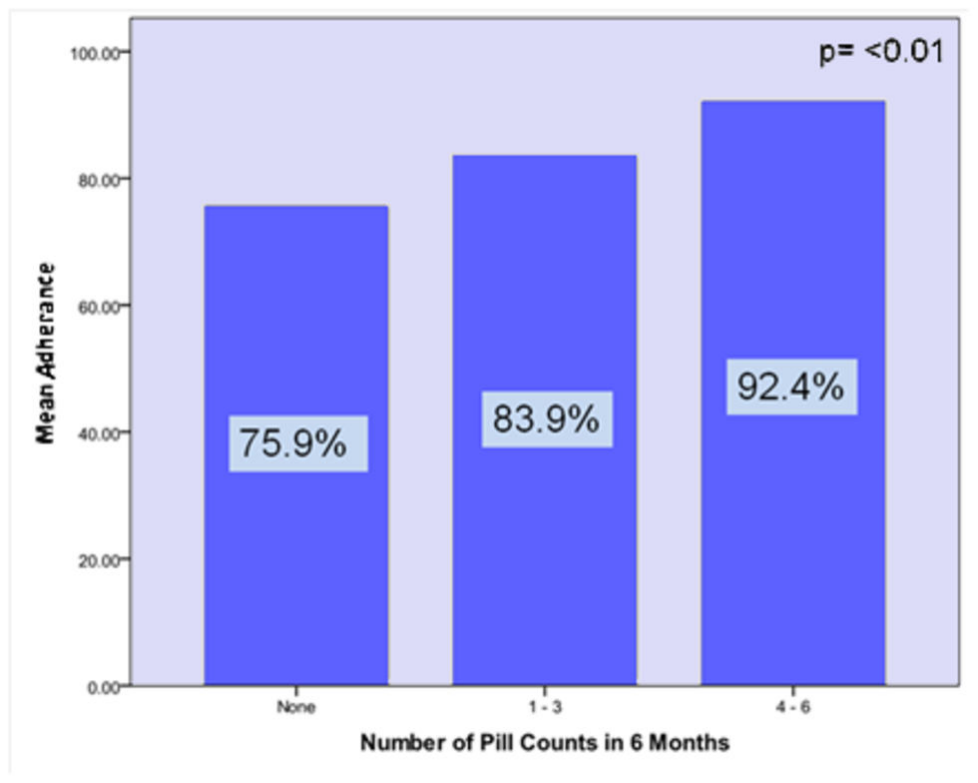
Mean Adherence by Pill Counts Category. Mean adherence in each of the three categories of pill counts performed showing increased adherence as the number of pill counts increased.

Figure 1 compares the percent adherence by number of pill counts performed. There was a significant increase in adherence based on pill count category with the lowest adherence in those having no pill counts performed (76%) and the highest in those having 4-6 pill counts performed (92%) (P=<0.01). Regression analysis demonstrates a linear relationship between the number of pill counts performed and patient adherence (r = .21, P< 0.01). Variance inflation factor (vif) for collinearity between clinician pill counts and clinic visits was 1.5. A vif of less than 5 suggests a lack of collinearity.

### Time to treatment failure

**Figure 2:**
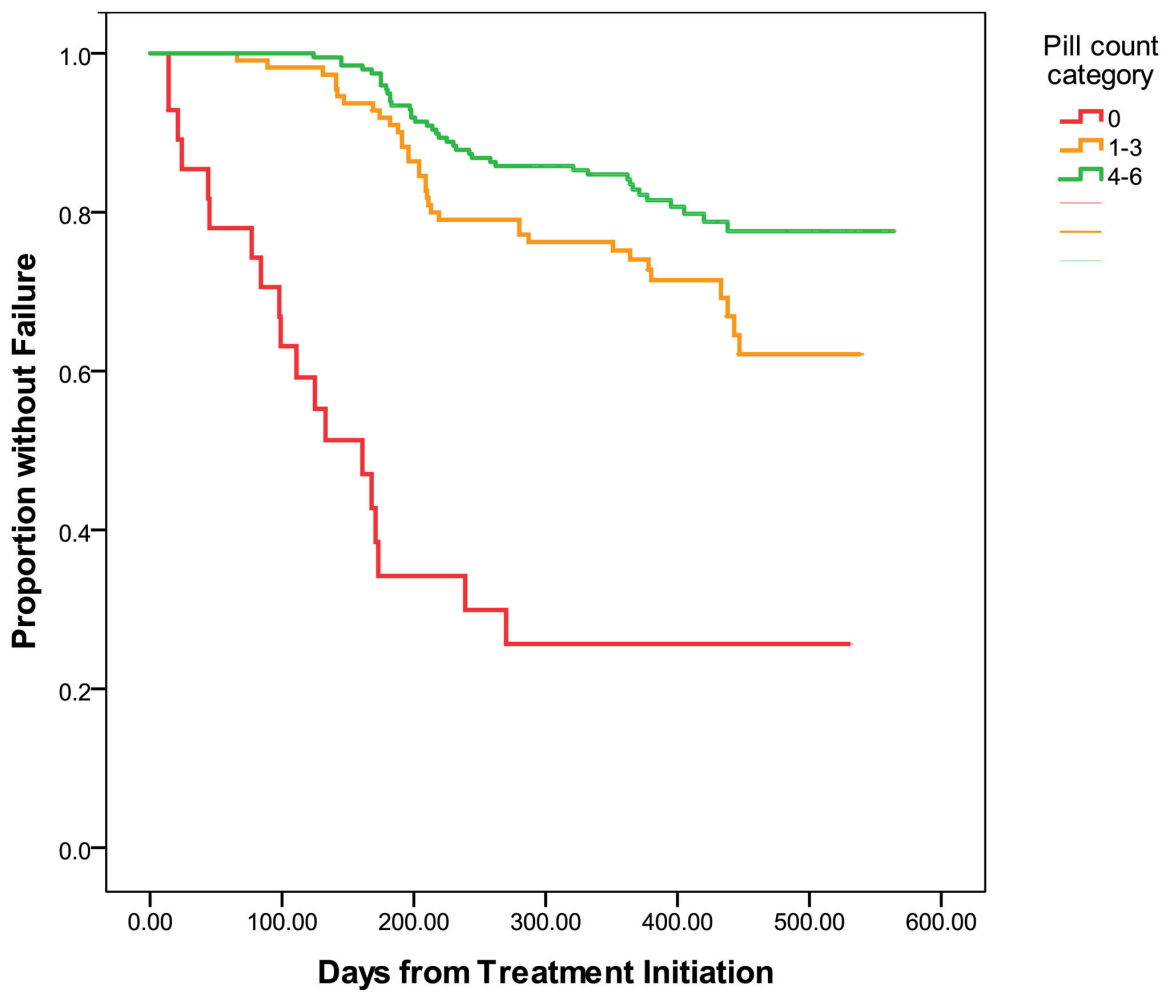
Time to Treatment Failure by Number of Pill Counts Performed. Time to treatment failure increased as the number of pill counts increased (none = 220 days, 1-3 = 438 days and = 497 days, P < 0.01).

Time to treatment failure for the primary end point is shown in Figure 2. There was a significantly longer time to treatment failure in those with more pill counts performed (none = 220 days, 1-3 = 438 days and = 497 days, P< 0.01). Time to virologic failure for those who were alive and in care demonstrated similar results (none = 296 days, 1-3 = 448 days and = 557 days, P = 0.02)

### Multivariate analysis

Multivariate analysis of the time to treatment failure using the Cox Proportional Hazards model is shown in Table 2. The baseline characteristics included in the model were baseline CD_4_ count, gender, and number of home visits, clinic visits, and support groups. Tests for two-way interaction between clinic visits and pill counts was not significant (p=0.19). On multivariate analysis, there was a significant increase in treatment success associated with total number of pill counts performed (HR = 0.69, P= 0.042) when adjusted for other factors. 

**Table 2:** Cox Hazard Ratios per program variable or intervention.

**FACTOR**	**Hazards Ratio**	**95% CI**	**P Value**
Number of Pill Counts	0.69	0.48- 0.98	.042
Gender	0.63	0.41- 0.99	.045
Support Groups (> 3)	0.76	0.46 -1.24	.274
Clinic Site	0.81	0.48- 1.36	.418
Number of Clinic Visits	0.70	0.60- 0.83	.000

## Discussion

In the current study we found an association between the number of clinician initiated pill counts preformed and subsequent patient outcomes. We observed that incremental increases in the number of pill counts preformed were associated improved adherence. More importantly, we found a strong association between the number of pill counts preformed and treatment success. Adherence interventions at Kijabe included both home visits and pharmacy counseling, each of which including a pill count by either a community health worker or a pharmacist. In our previous study, we found that only the pill count by the clinician who was providing direct HIV medical care was associated with a better outcome. In addition, clinician pill counts were the only activity that was discretionary and un-announced. Adjusting for participation in other adherence related activities, clinician initiated pill counts provided for the strongest association with better adherence and outcomes. 

By their nature, observational cohorts have un-measured biases that affect outcomes. A major concern is a selection bias in choosing which patients would have a pill count. Clinician pill counts were performed at the discretion of the provider. There are no incentives for performing pill counts no penalties for not performing pill counts. Patients who had more than three pill counts performed had a lower average CD4 cell count than those who did not, suggesting that clinicians were more attentive to sicker patients who would likely have a poorer outcome. Women were more likely to have pill counts performed and had better outcomes than men. This is probably reflective of the fact that women comprise the majority of HIV patients in care in sub-Saharan Africa. Finally, we did not see any difference in pill counts based on the specific clinic site where the patient received care. Despite careful analysis, we were unable to determine any pattern as to why a particular patient would have a pill count performed. The most likely explanation is that the clinics were busy and the clinicians were not able to count the pills for every patient. Despite this, the overall performance was good, with pill counts being completed in 68% percent of encounters. 

A second major concern in this study is that the patients who came to clinic were most likely to have clinician pill counts performed and thus, the association between pill counts and outcomes is in fact, due to co-linearity between pill counts and clinic visits. We believe that this is not the case for several reasons. These pill counts were performed at 68% of the 1800 clinic visits that occurred during the follow-up period, creating an environment where we could independently test the effects of each on the outcomes. In addition, there was no evidence of an interaction between clinician pill counts and clinic visits on regression analysis. A similar concern is that subjects who died or were lost to follow up will not have as many pill counts performed. To test this possibility, we performed a secondary analysis of only those patients who were alive and in care at the end of the study. In this group, we also found that the time to virologic failure was better in those who had more pill counts performed. 

It is also possible that there are other un-identified biases may also affect the outcome. There may have been a selection bias on the part of the clinician. It is possible that patients may have discarded their pills prior to their visits because they were worried that their doctor may count their pills. Our study had no mechanism to measure this possibility. Never the less, such actions on the part of the patients would bias the results toward showing no difference in outcome based on clinician pill counts. Finally, the study was conducted at a clinic system in rural Kenya and thus results may not be generalizable.

Despite these concerns, we believe that clinician pill counts may offer a simple and cost-effective intervention to improve patient outcomes in resource limited setting. Pill counts are easy to perform and provide tangible evidence to both provider and patient of adherence. They can be quickly performed without adding significant time to the visit. In resource limited settings, pill counts are an integral part of the provider-patient encounter and may mediate patient adherence to therapy [13,14]. On the other hand, two studies performed in the United States did not show a relationship between pill counts and adherence [15,16]. However these studies did not examine the effects of pill counts performed by physicians. 

We believe the role of pill counts by clinicians merits more study. The best way to resolve the questions regarding the utility of pill counts would be through a randomized study. Such a design would avoid many of the potential biases that are inherent in observational studies. It would address whether pill counts are a confounder in studies where they are used as a metric of adherence. A positive benefit from pill counting in resource limited settings would warrant including this intervention into routine clinical care of HIV patients. 
